# In silico investigation of mitragynine and 7-hydroxymitragynine metabolism

**DOI:** 10.1186/s13104-019-4461-3

**Published:** 2019-07-22

**Authors:** Taweetham Limpanuparb, Rattha Noorat, Yuthana Tantirungrotechai

**Affiliations:** 1 Science Division, Mahidol University International College, Mahidol University, Nakhon Pathom, 73170 Thailand; 2 Division of Chemistry, Faculty of Science and Technology, Thammasat University, Pathum Thani, 12120 Thailand

**Keywords:** Density functional theory, Kratom, Metabolism, Mitragynine, 7-Hydroxymitragynine

## Abstract

**Objective:**

Mitragynine is the main active compound of *Mitragyna speciose* (Kratom in Thai). The understanding of mitragynine derivative metabolism in human body is required to develop effective detection techniques in case of drug abuse or establish an appropriate dosage in case of medicinal uses. This in silico study is based upon in vivo results in rat and human by Philipp et al. (J Mass Spectrom 44:1249–1261, 2009).

**Results:**

Gas-phase structures of mitragynine, 7-hydroxymitragynine and their metabolites were obtained by quantum chemical method at B3LYP/6-311++G(d,p) level. Results in terms of standard Gibbs energies of reaction for all metabolic pathways are reported with solvation energy from SMD model. We found that 7-hydroxy substitution leads to changes in reactivity in comparison to mitragynine: position 17 is more reactive towards demethylation and conjugation with glucuronic acid and position 9 is less reactive towards conjugation with glucuronic acid. Despite the changes, position 9 is the most reactive for demethylation and position 17 is the most reactive for conjugation with glucuronic acid for both mitragynine and 7-hydroxymitragynine. Our results suggest that 7-hydroxy substitution could lead to different metabolic pathways and raise an important question for further experimental studies of this more potent derivative.

**Electronic supplementary material:**

The online version of this article (10.1186/s13104-019-4461-3) contains supplementary material, which is available to authorized users.

## Introduction

Mitragynine is the alkaloid derived from Kratom (*Mitragyna speciose*), a plant commonly found in Thailand and throughout the South East Asia region [1, 2]. Like many other opioid plants, there are claims of medical uses [3, 4] but these plants are also potentially illegal drugs of abuse [5, 6]. In additional to natural sources, mitragynine and its derivative may be obtained by total syntheses reported by researchers in Japan [7] and United States [8]. One of the more potent but naturally occurring derivatives of mitragynine is 7-hydroxymitragynine [9]. To establish an appropriate dosage in case of medicinal uses or to develop detection techniques in case of drug abuse, the understanding of mitragynine derivative metabolism in human body is needed.

**Fig. 1:**
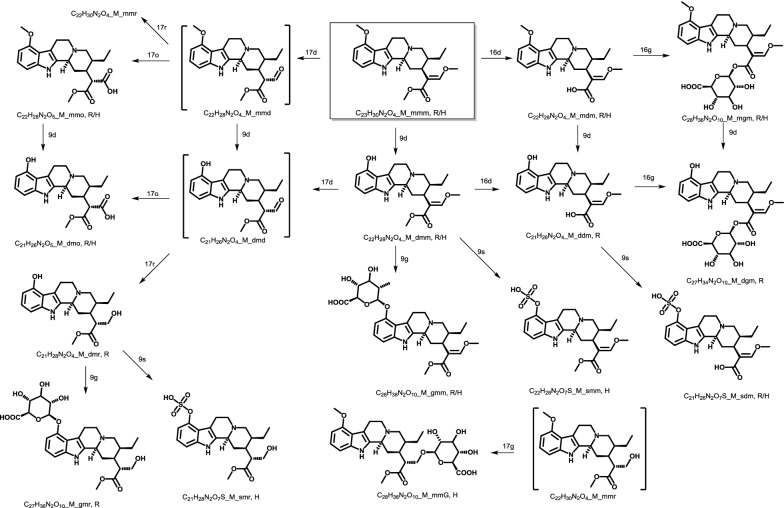
Metabolic pathways of mitragynine in rat and human was proposed by Philipp et al. [11] from LC-MS experiments in 2009 (Refer to Table 1 for abbreviated name convention. Compounds in brackets are not found experimentally but are assumed to be intermediates. Left, right and down arrows are for reactions at positions 17, 16 and 9 respectively. Metabolites found in rat or human are indicated by R or H respectively)

There were a number of experimental attempts to investigate metabolites of mitragynine and similar compounds in living organisms [10–12]. The most complete metabolic pathways of mitragynine were proposed from LC–MS study of rats and human urine samples by Philipp et al. [11] (see Fig. 1). Two different sample preparation techniques were used in Phase I and II metabolite extraction from urine. Missing intermediates were proposed and differences between compounds found in human and rats were attributed to physiological difference and/or differences in dosage/sampling time. The chemical reactions involved in the metabolic pathways are

- Hydrolysis of the only methyl ester at position 16,
- *O*-demethylation of the 9-methoxy group,
- *O*-demethylation of the 17-methoxy group, which may proceed via an aldehyde intermediate and result in corresponding carboxylic acid or alcohol by oxidation or reduction respectively,
- Subsequent conjugation with glucuronic acid at one of the three position above,
- Subsequent conjugation with sulfate only at the 9-methoxy group, and
- Combination of the steps above.

Figure 1 is the basis of this study and our aim is to complement the experimental findings with results from quantum chemical calculation. For comparison purpose, 7-hydroxymitragynine, a representative of naturally occurring and more potent derivatives of mitragynine is also included in our in silico investigation.

Conformers of mitragynine derivatives were theoretically studied by Liu et al. [13] using MMFF94s force field and B3LYP density functional theory method. Lowest energy structures were confirmed with crystal structures [14–17]. We used the lowest energy structures shown in Additional file 2: Figure S1 as the representative for our metabolic study. According to the figure, from left to right, the first and second rings are planar because of their aromaticity but the third and the fourth rings are both in chair conformer. The nitrogen atom between the third and fourth rings is above the plan of the molecule as described by Liu et al. [13].

## Main text

All quantum chemical calculations were performed using the Q-Chem 5.1 program package [18]. Gas-phase structures were obtained at B3LYP/6-311++G(d,p) level and were confirmed to be minima on the electronic potential energy surface by frequency calculations. Solvation energy in water from SMD model [19] was obtained from gas-phase geometries. The energies were corrected to standard state in solution condition of 1 M at 298.15 K with an exception of water in which case 55.34 M was used. Shell script, spreadsheet template and Mathematica [20] notebook are modified from our previous work [21]. All output files and other associated codes to obtain the standard Gibbs energies of reaction are provided in Additional file 1. For reporting purpose, metabolites are coded by molecular formula and abbreviated names as shown in Fig. 1 and Table 1.

**Table 1:** Abbreviated compound names used in this study

Parent compound	Position 9 (methyl ether of benzene)	Position 16 (methyl ester of alkene)	Position 17 (methyl ether of alkene)
M for mitragynine H for 7-hydroxymitragynine	m for original methyl group d for demethylation g for glucuronidation after d s for sulfation after d 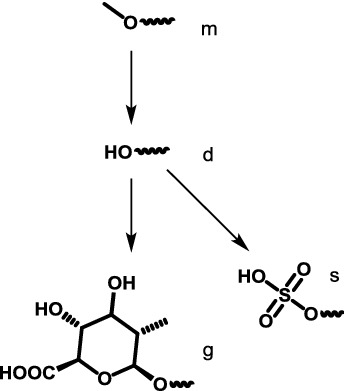 (4 possible groups and 3 possible steps)	m for original methyl group d for demethylation g for glucuronidation after d 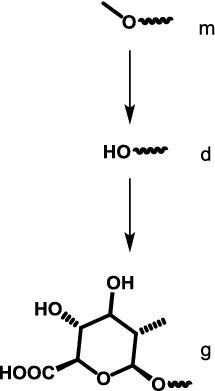 (3 possible groups and 2 possible steps)	m for original methyl group d for demethylation o for oxidation after d r for reduction after d G for glucuronidation after r 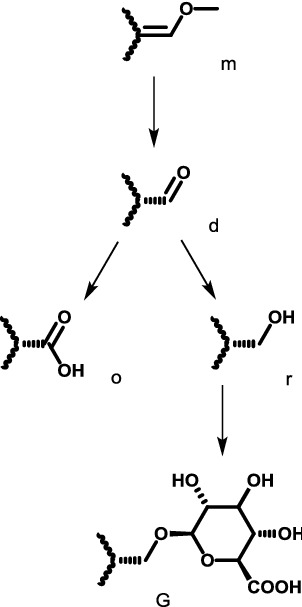 (5 possible groups and 4 possible steps)

Permutations of all possible substitutions at position 9 (m,d,g,s), position 16 (m,d,g) and position 17 (m,d,o,r,G) leads to 4 × 3 × 5 = 60 possible metabolites, and (3 × 3 × 5) + (4 × 2 × 5) + (4 × 3 × 4) = 133 possible steps. However, here we only consider compounds shown in Fig. 1 (18 structures and 21 possible steps) and their 7-hydroxy derivatives.

To compute reaction energy from Fig. 1, relevant additional reagents (water, sulfate ion, hydronium ion, protonated nicotinamide [22], glucuronic acid, oxygen) and products (water, methanol, reduced nicotinamide) were also added to the scheme to complete the thermodynamic calculation. These compounds are commonly found in biological systems and are likely to be reasonable energy reference point for these demethylation, oxidation/reduction of aldehyde and conjugation with glucuronide/sulfate reactions.

All calculations were completed with no imaginary frequency. The lowest energy structures of mitragynine and 7-hydroxymitragynine are shown in Additional file 2: Fig. S1. The detailed result for all reactions is listed in Additional file 2: Table S1 by type of reaction, number of steps from the parent compound and by position of reaction (Additional files 1, 2)

**Fig. 2:**
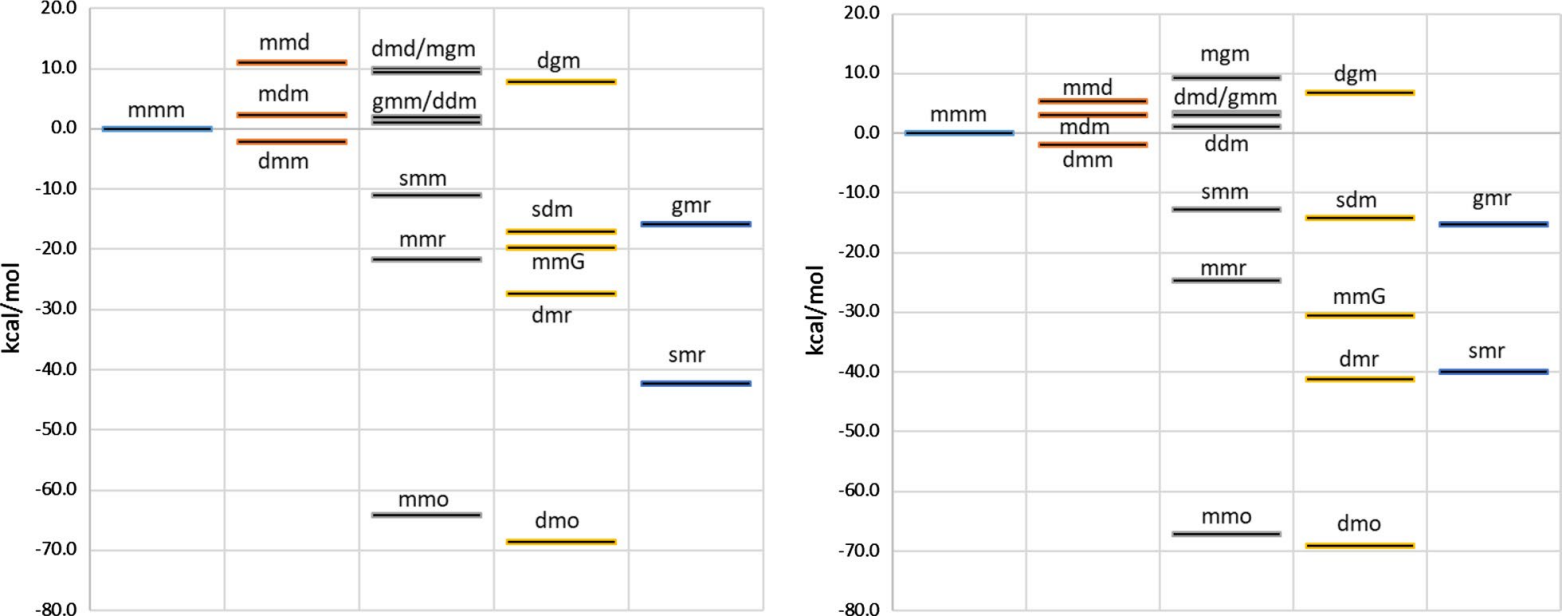
Relative standard Gibbs energies of metabolites of mitragynine (left) and 7-hydroxymitragynine (right) with reference to parent compounds, M_mmm and H_mmm. The energy of H_mmm is lower than that of M_mmm by 38.3 kcal/mol

- Gas-phase Gibbs energies of reaction show similar trend to aqueous-phase energies. The differences between gas-phase and aqueous-phase energies for conjugation with sulfate and reduction reactions are considerably large due to the presence of charged species on the reactant and/or product sides.
- The major determining factor for the energy of reaction is the type of reaction. Conjugation with sulfate, oxidation and reductions are highly exergonic and should occur easily. However, demethylation and conjugation with glucuronic acid have mixed results. The positions therefore play an important role in these cases to determine which pathway is more energetically favorable.
- The average standard Gibbs energies of reaction for each reaction-position pair is shown in Additional file 2: Figure S2. As far as the three positions are concerned, position 9 is the most reactive for demethylation and position 17 is the most reactive for conjugation with glucuronic acid.
- There are no conclusive trends from the number of steps from the parent compound.
- The effect of 7-hydroxy substitution can be seen in Additional file 2: Figure S2 (decreases in d17, g17 and an increase in g9 average reaction energies) and Fig. 2 (lower position of H_mmd, H_dmd and H_mmG, significantly lower position of H_dmr and slightly higher position of H_gmr). Despite the decreases and increase, the general conclusion above that position 9 is the most reactive for demethylation and position 17 is the most reactive for conjugation with glucuronic acid are still true for both mitragynine and 7-hydroxymitragynine.

Based on existing experimental evidence [11, 16], gas-phase structures of mitragynine, 7-hydroxymitragynine and their metabolites (in total 36 compounds) were obtained by quantum chemical method at B3LYP/6-311++G(d,p). Standard Gibbs energies of reaction in solution-phase were calculated by SMD solvation model. Four factors, type of reaction, number of steps from the parent compound, position of reaction and 7-hydroxy substitution effect were studied in this work. When compared to mitragynine, the 7-hydroxy substitution makes demethylation and conjugation with glucuronic acid at position 17 more favorable and makes conjugation with glucuronic acid at position 9 less favorable. Our results can be a basis for further experimental investigation of physiological effects of the compounds and/or detection of Kratom use.

## Limitations

During the preparation of this paper, relevant work [23–25] emerged in the literature. Readers may be interested to see a recent in vivo pharmacokinetics study of mitragynine in rats [23] and in vitro conversion of mitragynine to 7-hydroxymitragynine [24, 25]. The importance of 7-hydroxymitragynine was raised as there is clear evidence that it can be formed by in vivo metabolism. This is different from the basic premise in this manuscript that 7-hydroxymitragynine is obtained from Kratom as a minor alkaloid component. However, this fact does not materially change our conclusion in the current study. Also, cytochrome P450 enzymes were identified as the key mediator of the process [23, 25] and molecular docking studying could provide a further insight into the process.

A recent paper [23] describes a putative metabolic pathways from mitragynine to 14 metabolites. Unlike [11] which we used as a basis of our study, structures of all metabolites except one (Met2) are not available in the paper. Metabolites in the paper [23] can be comparable to ours as follow: Met5 is M_dmd, Met2 is possibly H-mmm, Met1 is possibly H_dmd, and Met8 is possibly M_mmd or M_mdm.

## Additional files

**Additional file 1.** All output files and other associated codes to obtain the standard Gibbs energies of reaction are provided.

**Additional file 2.** Table S1, Figures S1 and S2 for the molecular structures and energies of reaction.

## Data Availability

All data generated or analysed during this study are included in this published article and its additional files.
