# Supplementary material for: In silico investigation of mitragynine and 7-hydroxymitragynine metabolism
Source: BMC Res Notes. 2019 Jul 22;12:451. doi: 10.1186/s13104-019-4461-3 (PMC6647094; doi:10.1186/s13104-019-4461-3)
Supplement: Supplementary file 2 — Additional file 2. Table S1, Figures S1 and S2 for the molecular structures and energies of reaction. [file 13104_2019_4461_MOESM2_ESM.pdf]

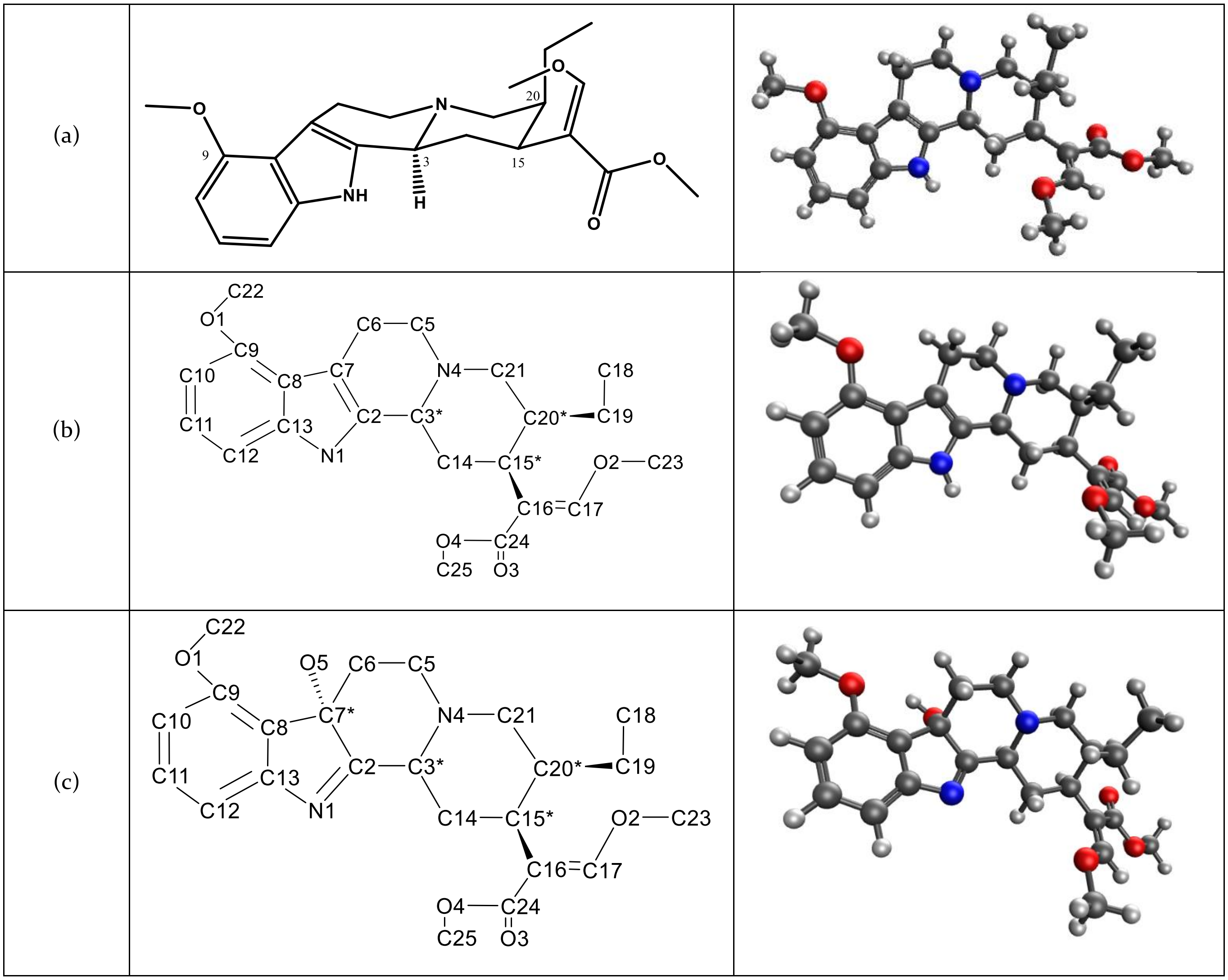

FIGURE 1S (a) Structure of mitragynine by Carvalho *et al.*(16) (b) Calculated lowest-energy structure of mitragynine (M_mmm) and (c) Calculated lowest-energy structure of 7-hydroxymitragynine (H_mmm)

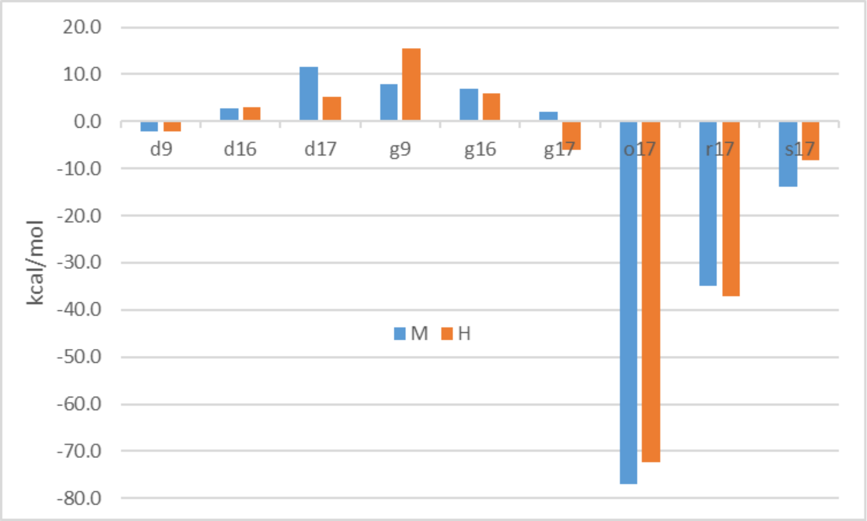

FIGURE 2S Average standard Gibbs energies of reaction for demethylation (d), conjugation to a glucuronide (g), oxidation (o), reduction (r), conjugation to sulfate (s) at different positions in mitragynine (M) and 7-hydroxymitragynine (H).

TABLE 1S Standard Gibbs energies of reaction for Mitragynine and 7-hydroxymitragynine metabolic pathways

Reactions	ΔG_{soln} kcal/mol	ΔG_{gas} kcal/mol	Step Number	Reaction	Position
d					
M_mmm + H_2O → CH_4O + M_dmm	-2.2	0.3	1	d	9
M_mmm + H_2O → CH_4O + M_mdm	2.3	8.3	1	d	16
M_mmm + H_2O → CH_4O + M_mmd	11.0	16.6	1	d	17
M_mdm + H_2O → CH_4O + M_ddm	-1.2	1.6	2	d	9
M_mmd + H_2O → CH_4O + M_dmd	-1.1	1.6	2	d	9
M_dmm + H_2O → CH_4O + M_ddm	3.3	9.6	2	d	16
M_dmm + H_2O → CH_4O + M_dmd	12.1	17.8	2	d	17
M_mmo + H_2O → CH_4O + M_dmo	-4.4	-4.8	3	d	9
M_mgm + H_2O → CH_4O + M_dgm	-1.6	0.4	3	d	9
H_mmm + H_2O → CH_4O + H_dmm	-1.9	-0.6	1	d	9
H_mmm + H_2O → CH_4O + H_mdm	3.0	8.9	1	d	16
H_mmm + H_2O → CH_4O + H_mmd	5.3	10.2	1	d	17
H_mdm + H_2O → CH_4O + H_ddm	-1.9	-0.5	2	d	9
H_mmd + H_2O → CH_4O + H_dmd	-1.9	-0.1	2	d	9
H_dmm + H_2O → CH_4O + H_ddm	3.0	9.0	2	d	16
H_dmm + H_2O → CH_4O + H_dmd	5.2	10.8	2	d	17
H_mmo + H2O → CH_4O + H_dmo	-2.0	-0.3	3	d	9
H_mgm + H_2O → CH_4O + H_dgm	-2.5	0.1	3	d	9
g					
M_dmm + $C_6H_{10}O_7$ → H_2O + M_gmm	4.2	-1.7	2	g	9
M_mdm + $C_6H_{10}O_7$ → H_2O + M_mgm	7.1	-1.1	2	g	16
M_ddm + $C_6H_{10}O_7$ → H_2O + M_dgm	6.6	-2.3	3	g	16
M_mmr + $C_6H_{10}O_7$ → H_2O + M_mmG	2.0	-4.2	3	g	17
M_dmr + $C_6H_{10}O_7$ → H_2O + M_gmr	11.6	6.9	4	g	9
H_dmm + $C_6H_{10}O_7$ → H_2O + H_gmm	4.9	3.4	2	g	9
H_mdm + $C_6H_{10}O_7$ → H_2O + H_mgm	6.3	-1.4	2	g	16
H_ddm + $C_6H_{10}O_7$ → H_2O + H_dgm	5.7	-0.8	3	g	16
H_mmr + $C_6H_{10}O_7$ → H_2O + H_mmG	-6.0	-14.6	3	g	17
H_dmr + $C_6H_{10}O_7$ → H_2O + H_gmr	26.0	21.6	4	g	9
s					
M_dmm + $2H_3O^+$ + SO_4^{2-} → $3H_2O$ + M_smm	-8.8	-409.6	2	s	9
M_ddm + $2H_3O^+$ + SO_4^{2-} → $3H_2O$ + M_sdm	-18.2	-423.9	3	s	9
M_dmr + $2H_3O^+$ + SO_4^{2-} → $3H_2O$ + M_smr	-14.9	-421.5	4	s	9
H_dmm + $2H_3O^+$ + SO_4^{2-} → $3H_2O$ + H_smm	-10.8	-412.7	2	s	9
H_ddm + $2H_3O^+$ + SO_4^{2-} → $3H_2O$ + H_sdm	-15.3	-419.6	3	s	9
H_dmr + $2H_3O^+$ + SO_4^{2-} → $3H_2O$ + H_smr	1.3	-401.9	4	s	9
r					
M_mmd + $C_6H_8N_2O$ + H_3O^+ → $C_6H_7N_2O^+$ + H_2O + M_mmr	-32.7	-67.4	2	r	17
M_dmd + $C_6H_8N_2O$ + H_3O^+ → $C_6H_7N_2O^+$ + H_2O + M_dmr	-37.3	-70.5	2	r	17
H_mmd + $C_6H_8N_2O$ + H_3O^+ → $C_6H_7N_2O^+$ + H_2O + H_mmr	-29.9	-61.8	2	r	17
H_dmd + $C_6H_8N_2O$ + H_3O^+ → $C_6H_7N_2O^+$ + H_2O + H_dmr	-44.5	-80.0	2	r	17
o					
M_mmd + $1/2O_2$ → M_mmo	-75.2	-70.0	2	o	17
M_dmd + $1/2O_2$ → M_dmo	-78.6	-76.3	2	o	17
H_mmd + $1/2O_2$ → H_mmo	-72.4	-69.9	2	o	17
H_dmd + $1/2O_2$ → H_dmo	-72.4	-70.1	2	o	17
